# The Mechanical Power in Patients with Acute Respiratory Distress Syndrome Undergoing Prone Positioning Can Predict Mortality

**DOI:** 10.3390/diagnostics15020158

**Published:** 2025-01-12

**Authors:** Ko-Wei Chang, Shaw-Woei Leu, Han-Chung Hu, Ming-Cheng Chan, Shinn-Jye Liang, Kuang-Yao Yang, Li-Chung Chiu, Wen-Feng Fang, Chau-Chyun Sheu, Ying-Chun Chien, Chung-Kan Peng, Ching-Tzu Huang, Kuo-Chin Kao

**Affiliations:** 1 Department of Thoracic Medicine, Chang Gung Memorial Hospital, Taoyuan 333, Taiwan; b9302072@cgmh.org.tw (K.-W.C.); swleu@cgmh.org.tw (S.-W.L.); h3226@cgmh.org.tw (H.-C.H.); cremaster54@yahoo.com.tw (L.-C.C.); 2 Graduate Institute of Clinical Medical Sciences, College of Medicine, Chang Gung University, Taoyuan 333, Taiwan; 3 School of Medicine, National Tsing Hua University, Hsinchu 300, Taiwan; 4 Department of Respiratory Therapy, College of Medicine, Chang Gung University, Taoyuan 333, Taiwan; e22110@cgmh.org.tw; 5 Division of Critical Care and Respiratory Therapy, Department of Internal Medicine, Taichung Veterans General Hospital, Taichung 407, Taiwan; mingcheng.chan@gmail.com; 6 College of Science, Tunghai University, Taichung 407, Taiwan; 7 Division of Pulmonary and Critical Care, Department of Internal Medicine, China Medical University Hospital, Taichung 404, Taiwan; reagon6142@gmail.com; 8 Department of Chest Medicine, Taipei Veterans General Hospital, Taipei 112, Taiwan; kyyang@vghtpe.gov.tw; 9 Institute of Emergency and Critical Care Medicine, School of Medicine, National Yang-Ming University, Taipei 112, Taiwan; 10 Division of Pulmonary and Critical Care Medicine, Department of Internal Medicine, Kaohsiung Chang Gung Memorial Hospital, Kaohsiung 833, Taiwan; wenfengfang@yahoo.com.tw; 11 Department of Respiratory Care, Chang Gung University of Science and Technology, Chiayi 613, Taiwan; 12 Division of Pulmonary and Critical Care Medicine, Department of Internal Medicine, Kaohsiung Medical University Hospital, Kaohsiung 807, Taiwan; sheu@kmu.edu.tw; 13 Department of Internal Medicine, School of Medicine, College of Medicine, Kaohsiung Medical University, Kaohsiung 807, Taiwan; 14 Division of Chest Medicine, Department of Internal Medicine, National Taiwan University Hospital, Taipei 100, Taiwan; firstaidg@gmail.com; 15 Division of Pulmonary and Critical Care Medicine, Department of Internal Medicine, Tri-Service General Hospital, National Defense Medical Center, Taipei 114, Taiwan; kanpeng@mail.ndmctsgh.edu.tw; 16 Department of Respiratory Therapy, Chang Gung Memorial Hospital, Taoyuan 333, Taiwan

**Keywords:** mechanical power, prone positioning, acute respiratory distress syndrome

## Abstract

**Background/Objectives:** Mechanical power (MP) refers to ventilator-delivered energy to the lungs, which may induce lung injury. We examined the relationship between MP and mortality in patients with acute respiratory distress syndrome (ARDS) who underwent prone positioning. **Methods:** This multicenter retrospective study included data on all patients admitted to the intensive care units of eight referral hospitals in Taiwan from October 2015 to March 2016, and in Chang Gung Memorial Hospital Linkou branch from January 2017 to October 2023. The data were obtained from the electronic medical records of each hospital by using a standard case report form. MP was calculated as follows: MP (J/min) = 0.098 × VT × RR × (Ppeak − 1/2 × ΔP). **Results:** We included 135 patients who underwent prone positioning. Among them, 28-day survivors had significantly lower MP (22.6 ± 6.5 vs. 25.3 ± 6.2 J/min, *p* = 0.024), MP/predicted body weight (PBW) (396.9 ± 118.9 vs. 449.3 ± 118.8 10^−3^ J/min/kg, *p* = 0.018), MP/compliance values (0.8 ± 0.3 vs. 1.1 ± 0.4 J/min/mL/cmH_2_O, *p* = 0.048) after prone positioning, and significantly lower changes in MP, MP/PBW, and MP/compliance (−0.6 ± 5.7 vs. 2.5 ± 7.4 J/min, *p* = 0.007; −9.2 ± 97.5 vs. 42.1 ± 127.9 10^−3^ J/min/kg, *p* = 0.010; −0.1 ± 0.3 vs. 0.2 ± 0.3 J/min/mL/cmH_2_O, *p* < 0.001, respectively). Multivariate Cox regression revealed that the change in MP/compliance (HR: 7.972, *p* < 0.001) was an independent predictive factor for 28-day mortality. **Conclusions:** In ARDS patients treated with prone positioning, MP/compliance, and change in MP, MP/PBW, and MP/compliance after prone positioning differed significantly between 28-day survivors and nonsurvivors. Further randomized controlled research is required to elucidate the potential causality of decreased MP and improved clinical outcomes.

## 1. Introduction

Acute respiratory distress syndrome (ARDS), defined by the Berlin definition [1], is a form of noncardiogenic pulmonary edema with bilateral pulmonary infiltration and arterial hypoxemia. Although mechanical ventilation is the cornerstone of treatment for hypoxemia, it may induce further lung injury [2]; gas delivery to the lungs through a ventilator may also lead to the transfer of a type of energy called mechanical power (MP) [3], which, when calculated by tidal volume, airway pressure, and respiratory rate, may cause lung injury. Several studies have reported a relationship between MP and mortality in patients with ARDS [4,5], patients in critical care [6], or patients with extracorporeal membrane oxygenation (ECMO) [7]. Moreover, studies have reported that MP normalized to predicted body weight (PBW) [5] or lung compliance [8] had a higher predictive value concerning mortality.

Although several treatment strategies for ARDS have been explored, only low tidal volume [9] and prone positioning [10] have been confirmed as having survival benefits. In 1974, prone positioning was noted to produce improvements in oxygenation [11]. However, the survival benefits of prone positioning were not documented until 2013 in the PROSEVA study [10], in which patients with moderate to severe ARDS who underwent sufficiently long sessions of prone positioning and for whom strict protective lung strategies were employed had survival benefits.

The literature does not contain adequate research on MP with a focus on patients with ARDS receiving prone positioning treatment. In a physiologic study [12], they found that prone positioning could reduce lung total elastic and elastic static power, but they did not mention clinical outcomes. Although an MP study by Guerin et al. [4] used the data of the patients from the PROSEVA study, the authors did not indicate which patients underwent prone positioning. To elucidate the relationship between MP and mortality and fill the aforementioned research gap, the present study examined patients with ARDS who underwent prone positioning.

## 2. Materials and Methods

### 2.1. Patients and Data Collection

We collected patients from Taiwan Severe Influenza Research Consortium and medical intensive care units (ICUs) in Chang Gung Memorial Hospital Linkou branch in this study. The Taiwan Severe Influenza Research Consortium comprises eight referral hospitals throughout Taiwan (four in Northern Taiwan, two in Central Taiwan, and two in Southern Taiwan). We collected data for all patients who were admitted to the ICUs of these eight hospitals due to influenza pneumonia-related ARDS from October 2015 to March 2016 with a uniform case report form. Influenza was diagnosed through nasal or oral swabs, sputum samples, or bronchoalveolar lavage samples that were tested using a rapid test or polymerase chain reaction test. We also collected patients who were admitted to medical ICUs in Chang Gung Memorial Hospital Linkou branch, a tertiary medical center in Taiwan with 56 medical ICU beds, from January 2017 to October 2023. ARDS was diagnosed according to the Berlin definition [1]. All patients who underwent prone positioning were included in the analysis. Demographic data, laboratory data, ventilator setting data, and outcomes were obtained from the electronic medical records of each hospital by using a standard case report form. Severity was scored based on the Acute Physiology and Chronic Health Evaluation II (APACHE II) score [13], and Sequential Organ Failure Assessment (SOFA) score [14]. We collected the severity score and laboratory data at the day that the patients underwent prone positioning.

This study was approved by the local institutional review boards for human research at every hospital involved [Linkou and Kaohsiung Chang Gung Memorial Hospital IRB No. 201600632B0 (30 September 2016), National Taiwan University Hospital 201605036RIND, Taipei Veterans General Hospital 2016-05-020CC, Tri-Service General Hospital 1-105-05-086, Taichung Veterans General Hospital CE16093A, China Medical University Hospital 105-REC2-053[FR], and Kaohsiung Medical University Hospital KUMHIRB-E[I]-20170097), and Linkou Chang Gung Memorial Hospital IRB No. 202400652B0 (3 May 2024). This retrospective study was exempt from informed consent.

### 2.2. Ventilator Settings

An intensivist and a respiratory therapist established the mechanical ventilator settings according to the ARDSnet with lung protective strategy protocol [9]. The positive end-expiratory pressure (PEEP) was adjusted based on the lower PEEP/FiO_2_ combination table [15]. All patients in this study were administered mechanical ventilation in pressure-control mode. Dynamic driving pressure was calculated as peak airway pressure minus PEEP, and dynamic compliance was calculated as tidal volume divided by dynamic driving pressure.

We collected data on the ventilator settings and the corresponding arterial blood gas levels at 2 time points: (1) just before prone positioning, and (2) 1 day after prone positioning with the patient under a stable condition with the longest usage settings.

### 2.3. Prone Positioning

The on-duty intensivist administered prone positioning according to the physician’s preference and the experience of the unit or hospital. Unstable hemodynamic status was the main contraindication in these patients. Prone positioning was administered for more than 16 h each day according to the PROSEVA study protocol [10].

### 2.4. Definition of MP Derivation

According to the procedures of previous studies [3,5,7], MP was calculated as follows:

MP (J/min) = 0.098 × VT × RR × (Ppeak − 1/2 × ΔP)

MP normalized to PBW (10^−3^ J/min/kg) = MP/PBW

MP normalized to lung compliance (J/min/mL/cmH_2_O) = MP/compliance = MP/(VT/ΔP),

where VT represents tidal volume, RR represents respiratory rate, Ppeak represents peak airway pressure, and ΔP represents dynamic driving pressure.

In previous studies, peak airway pressure served as a surrogate for plateau pressure in the calculation of MP [7,16], and the mortality predictive value of MP was determined to be the same when peak airway pressure was used instead of plateau pressure [6]. One study referred to MP calculated using peak airway pressure as “dynamic MP” [17]. Changes in MP, MP/PBW, or MP/compliance were calculated through the subtraction of data obtained before prone positioning from those obtained after prone positioning.

### 2.5. Statistical Analysis

Continuous variables are presented as mean ± standard deviation; depending on the underlying distribution, the Student *t*-test or the Mann–Whitney U test was performed to compare the data. Nominal variables are presented as numbers (percentages); depending on the underlying distribution, the chi-squared test or Fisher’s exact test was used to compare the data. A paired *t*-test was used to compare the ventilator parameters before and after prone positioning. The area under the receiver operating characteristic curve and the C-statistic were used to analyze the predictive ability of different factors, and the Youden index was applied to determine the optimal cutoff value. Kaplan–Meier survival analysis and the log-rank test were used to analyze the survival conditions in different groups. We used the Cox regression model to determine the univariate and multivariate predictors of 28-day mortality, and factors with *p* < 0.01 in univariate analysis were selected for multivariate analysis. Two models with different types of MP were used in the multivariate analysis. Statistical significance was defined as *p* < 0.05. All statistical analyses were performed using SPSS (version 22.0; SPSS, Chicago, IL, USA).

## 3. Results

### 3.1. Demographic Data

**Figure 1:**
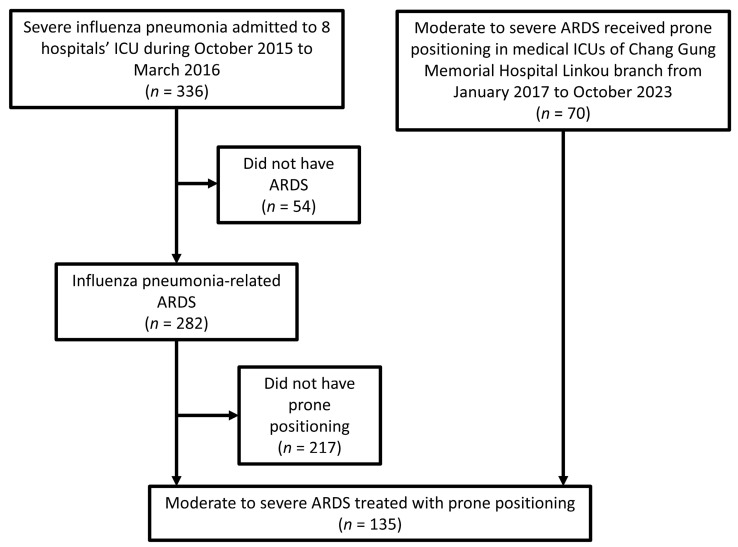
Flow chart of patients in this study; ARDS: acute respiratory distress syndrome; ICU: intensive care unit.

In total, 135 patients were included in this study, 65 patients were collected from Taiwan Severe Influenza Research Consortium and 70 patients were collected from medical ICUs in Chang Gung Memorial Hospital Linkou branch. The flow chart is presented in Figure 1. The etiology of ARDS was influenza in all 65 patients in Taiwan Severe Influenza Research Consortium. In the Chang Gung Memorial Hospital Linkou branch medical ICUs, 21 patients suffered from viral pneumonia (including influenza, coronavirus disease 2019, or cytomegalovirus), 43 patients suffered from bacterial pneumonia, and 6 patients suffered from sepsis from non-pulmonary infectious sources. There were 86 (63.7%) male patients, and the average age was 58.2 ± 13.0 years. Prone positioning was started at 1.7 ± 2.6 days, and the period of prone positioning administration was 3.5 ± 3.6 days. The PaO_2_/FiO_2_ ratio for all patients who underwent prone positioning was 87.5 ± 42.1 mm Hg.

Compared with the 28-day nonsurvivors, the 28-day survivors had significantly higher body mass index values (26.7 ± 5.8 vs. 23.4 ± 3.6 kg/m^2^, *p* < 0.001); less malignancy (8.8% vs. 29.5%, *p* = 0.002); lower APACHE II scores (24.9 ± 7.5 vs. 29.5 ± 5.5, *p* < 0.001), and SOFA scores (11.9 ± 3.0 vs. 13.6 ± 2.8, *p* = 0.003); lower C-reactive protein levels (15.4 ± 9.6 vs. 22.0 ± 11.3 mg/dL, *p* = 0.001); lower lactate levels (20.6 ± 18.7 vs. 31.0 ± 31.5 mg/dL, *p* = 0.046); lower creatinine levels (1.6 ± 2.1 vs. 2.8 ± 3.1 mg/dL, *p* = 0.004); higher PaO_2_/FiO_2_ ratio (93.3 ± 46.4 vs. 75.4 ± 28.3 mmHg, *p* = 0.020); and fewer patients requiring renal replacement therapy (19.8% vs. 43.2%, *p* = 0.004; Table 1). Extracorporeal membrane oxygenation was used as salvage therapy for prone positioning failure in 18 patients, and 7 patients died within 28 days.

### 3.2. Ventilator Parameters

We compared the ventilator parameters before and after prone positioning in all patients. The comparison revealed significantly higher PEEP (13.6 ± 2.9 vs. 14.2 ± 2.3 cmH_2_O, *p* = 0.005), lower dynamic driving pressure (18.9 ± 4.5 vs. 18.1 ± 4.6 cmH_2_O, *p* = 0.002), and higher respiratory rate (24.7 ± 4.7 vs. 25.5 ± 4.5/min, *p* = 0.040) after prone positioning was administered. No significant change was noted in dynamic lung compliance.

The 28-day survivors had significantly lower dynamic driving pressure before prone positioning (18.3 ± 3.9 vs. 20.2 ± 5.3 cmH_2_O, *p* = 0.035). Moreover, the 28-day survivors had significantly lower peak airway pressure (31.0 ± 3.7 vs. 34.9 ± 5.1 cmH_2_O, *p* < 0.001), lower dynamic driving pressure (16.8 ± 3.7 vs. 20.7 ± 5.3 cmH_2_O, *p* < 0.001) levels, higher dynamic lung compliance (25.7 ± 8.1 vs. 21.0 ± 8.5 mL/cmH_2_O, *p* = 0.02), and lower respiratory rate (24.9 ± 4.5 vs. 26.8 ± 4.3/min, *p* = 0.017) after prone positioning (Table 2).

### 3.3. MP Derivation

The MP, MP/PBW, and MP/compliance values before prone positioning did not differ significantly between the 28-day survivors and nonsurvivors. However, the 28-day survivors had significantly lower MP (22.6 ± 6.5 vs. 25.3 ± 6.2 J/min, *p* = 0.024), MP/PBW (396.9 ± 118.9 vs. 449.3 ± 118.8 10^−3^ J/min/kg, *p* = 0.018), and MP/compliance values after prone positioning (0.9 ± 0.3 vs. 1.4 ± 0.6 J/min/mL/cmH_2_O, *p* < 0.001) (Table 2). The statistical power levels for the MP, MP/PBW, and MP/compliance after prone positioning between the 28-day survivors and nonsurvivors were 0.620, 0.662, and 1.000, respectively. In all of the patients, the MP, MP/PBW, or MP/compliance values before prone positioning did not differ significantly from those after prone positioning (23.3 ± 6.2 vs. 23.6 ± 6.4 J/min, *p* = 0.558; 408.5 ± 116.3 vs. 414.5 ± 119.5 10^−3^ J/min/kg, *p* = 0.528; 1.1 ± 0.5 vs. 1.1 ± 0.5 J/min/mL/cmH_2_O, *p* = 0.738, respectively).

We also calculated changes in MP, MP/PBW, and MP/compliance before and 1 day after the first session of prone positioning (i.e., values derived 1 day after prone positioning minus those derived before prone positioning). The MP, MP/PBW, and MP/compliance values decreased after prone positioning in both the 28-day and the 60-day survivors but increased in the nonsurvivors; the values differed significantly between the 28-day and 60-day survivors and nonsurvivors (MP: −0.6 ± 5.7 vs. 2.5 ± 7.4 J/min, *p* = 0.007; −0.6 ± 5.8 vs. 1.8± 7.0 J/min, *p* = 0.008, respectively; MP/PBW: −9.2 ± 97.5 vs. 42.1 ± 127.9 10^−3^ J/min/kg, *p* = 0.010; −10.9 ± 97.6 vs. 31.8 ± 122.4 10^−3^ J/min/kg, *p* = 0.009, respectively; MP/compliance: −0.1 ± 0.3 vs. 0.2 ± 0.3 J/min/mL/cmH_2_O, *p* < 0.001; −0.1 ± 0.3 vs. 0.2 ± 0.3 J/min/mL/cmH_2_O, *p* < 0.001, respectively). The statistical power levels for the changes in MP, MP/PBW, and MP/compliance between the 28-day survivors and nonsurvivors were 0.748, 0.719, and 1.000, respectively.

**Figure 2:**
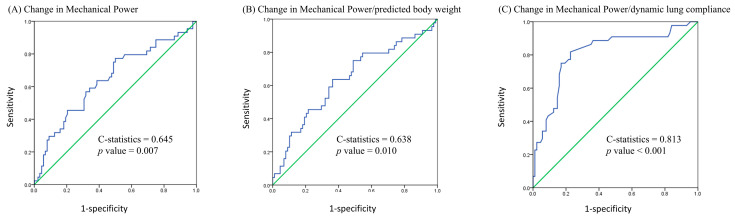
Receiver operating characteristic curve using 28-day mortality as the outcome of interest.

**Figure 3:**
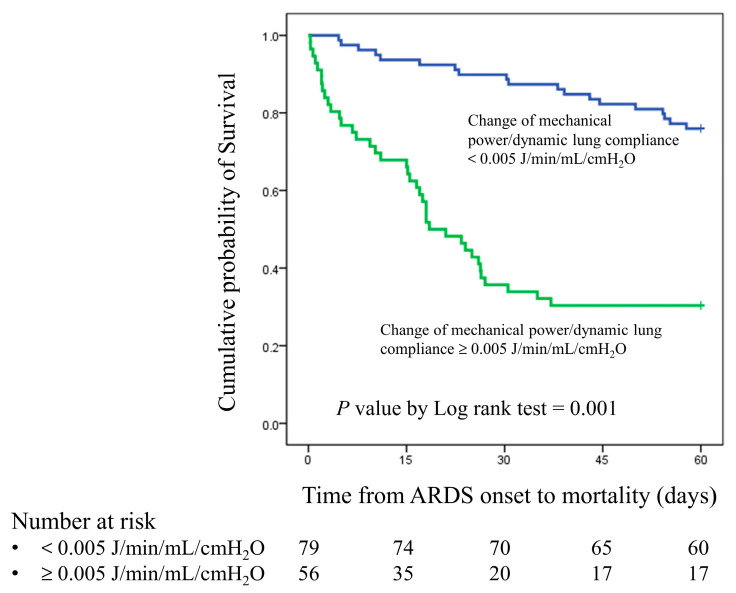
Kaplan–Meier survival curve for patients with a change in MP/compliance of ≥0.005 J/min/mL/cmH_2_O and patients with a change in MP/compliance of <0.005 J/min/mL/cmH_2_O.

We used a receiver operating characteristic curve to analyze the 28-day mortality predictive value of changes in MP, MP/PBW, and MP/compliance after prone positioning. The C-statistics for the changes in MP, MP/PBW, and MP/compliance were 0.645 (95% confidence interval [CI], 0.542–0.747, *p* = 0.007), 0.638 (95% CI, 0.535–0.741, *p* = 0.010), and 0.813 (95% CI, 0.730–0.896, *p* < 0.001), respectively (Figure 2). We also used Youden’s index to determine the optimal cutoff value for the change in MP/compliance, which was +0.005 J/min/mL/cmH_2_O (Youden’s index was 0.591). Patients with a change in MP/compliance of ≥0.005 J/min/mL/cmH_2_O had significantly higher 28-day and 60-day mortality rates than did those with a change of <0.005 J/min/mL/cmH_2_O (66.0% vs. 13.4%, *p* = 0.001; 72.0% vs. 26.8%, *p* = 0.001). The Kaplan–Meier curve is presented in Figure 3.

### 3.4. Univariate and Multivariate Cox Regression Analysis

In the univariate Cox regression analysis, the body mass index, APACHE II score, SOFA score, C-reactive protein levels, lactate levels, creatinine levels, PaO_2_/FiO_2_ ratio before prone positioning, PaO_2_/FiO_2_ ratio, PaCO_2_ levels, MP, MP/PBW, MP/compliance after prone positioning, change in MP, MP/PBW, and MP/compliance were significant predictors of 28-day mortality (Table 3). In the model 1 multivariate analysis involving MP/compliance after prone positioning, the body mass index (HR: 0.926, *p* = 0.046), PaO_2_/FiO_2_ ratio after prone positioning (HR: 0.991, *p* = 0.015), and MP/compliance after prone positioning (HR: 3.486, *p* = 0.006) were independent predictive factors. In the model 2 multivariate analysis involving the change in MP/compliance, the SOFA score (HR: 1.172, *p* = 0.034) and change in MP/compliance (HR: 7.972, *p* < 0.001) were independent predictive factors (Table 3).

## 4. Discussion

In this retrospective cohort study, we focused on patients with moderate to severe ARDS who received prone positioning. We observed that although MP did not change significantly 1 day after the first prone positioning, post-prone positioning MP (including MP/PBW and MP/compliance) and the change in MP (including MP/PBW and MP/compliance) after prone positioning were significantly correlated with 28-day mortality.

The survival benefit of prone positioning for patients with moderate to severe ARDS was reported in a previous study [10]; nevertheless, the exact mechanism underlying this effect remains unknown. Several physiological mechanisms have been proposed in previous studies, such as the homogenization of lung aeration [18,19]; reduction in shunt fraction [18,20]; reduction in ventilator-induced lung injury (VILI), including volutrauma [21], atelectrauma [19], and biotrauma [22]; reduction in lung compression by the heart [23] or abdomen [24]; and improvement of secretion clearance [25]. MP, meaning the energy that a ventilator delivers to the lung, may engender lung injury [3,26]. MP is a single parameter that includes several components of VILI, such as pressure, volume, flow, and respiratory rate [26]. Moreover, VILI may lead to multiorgan failure and death [2]. In our study, we found that survivors had significantly lower MP levels after prone positioning and exhibited a change in MP after prone positioning. This is likely because the first session of prone positioning may prevent MP and VILI from increasing, which may result in favorable clinical outcomes; hence, prone positioning leading to higher survival rates may be due to a decrease in VILI. If MP and VILI do not decrease in patients after prone positioning, the prone positioning may have failed and additional rescue strategies may be required.

However, studies have yet to report a causality between a decrease in MP and improved clinical outcomes. A decrease in MP may lead to a decrease in VILI [26]; a decrease in MP may also indicate an improvement in lung conditions, thereby reducing the level of ventilator support necessary. In the patients included in our study, dynamic lung compliance improved (from 24.0 ± 7.0 to 25.7 ± 8.1 mL/cmH_2_O) in 28-day survivors and worsened (from 22.6 ± 9.6 to 21.0 ± 8.5 mL/cmH_2_O) in nonsurvivors. Although tidal volume did not change significantly after prone positioning, the decrease in dynamic driving pressure might have resulted in a decrease in MP. Further randomized controlled research is required to elucidate any causality.

Mechanical power is typically calculated using a complex formula with several parameters, including plateau (peak) pressure, PEEP, tidal volume, and respiratory rate [3]. However, the order of importance for these parameters remains unclear [27,28]. Costa et al. considered only driving pressure and respiratory rate as independent predictive factors for survival [27]. In our previous study, we observed that changes in peak airway pressure and dynamic driving pressure before and after prone positioning were significantly correlated with survival [29]. This finding suggests that peak airway pressure or driving pressure is the most crucial MP parameter influencing improved survival in patients. However, in this study, the C-statistics for the change in dynamic driving pressure and survival and the change in MP/compliance and survival were 0.729 and 0.813, respectively, indicating that MP/compliance is a superior predictor of survival in patients with ARDS.

In this study, we found MP/compliance to be a superior predictor of survival compared with MP alone or MP/PBW in patients receiving prone positioning. This finding is similar to those of previous studies on patients with ARDS. Zhang et al. demonstrated that MP/PBW and MP/compliance on day 0 were superior predictors of mortality compared with MP alone (*p* = 0.011); however, no significant difference was noted between MP/PBW and MP/compliance (*p* = 0.659) [5]. Coppola et al. also revealed that MP alone did not differ significantly between survivors and nonsurvivors but that MP/compliance and MP/well-inflated tissue were independently associated with ICU mortality [8]. Chiu et al. focused on patients with severe ARDS who required ECMO as rescue therapy; they observed that post-ECMO MP/compliance (*p* < 0.001) had a higher predictive value for mortality than MP alone (*p* = 0.022) [7]. According to these studies, MP normalized to functional lung size is a more accurate reflection of the energy applied to the lungs [5,8], and functional lung size is more effectively quantified using compliance than PBW [30,31].

Our group’s previous study reported that MP decreased by 49% after the first day of ECMO and that only post-ECMO MP could predict mortality [7]. In our study, we also observed that MP, MP/PBW, and MP/compliance after prone positioning could predict mortality; however, MP did not change significantly after prone positioning. Although the decrease in MP after prone positioning was much smaller than that observed post-ECMO, MP after prone positioning and the change in MP before and after prone positioning could predict mortality.

This retrospective study has some limitations. First, the number of included patients was limited even though we collected patients from two cohorts. However, the statistical power was as high as 1.000, which may be sufficient to support our conclusion. Second, even though more than half of the patients were influenza pneumonia-related ARDS, we also included patients with different etiologies. Whether different etiologies may influence the results requires further research. Nevertheless, we consciously avoided the inclusion of patients with factors that might influence predictions of mortality, such as the administration of palliative care. Third, because ventilation was administered in pressure-controlled mode for all of the patients in our study, we could not obtain data on plateau pressure. However, several studies have reported peak airway pressure to be a potential surrogate for plateau pressure in calculating MP [16], with the peak airway pressure being almost equal to plateau pressure when the end-inspiratory flow is near zero [32]. Finally, this was a multicenter retrospective study; although all of the hospitals adhere to contemporary guidelines for treating patients, medical centers inevitably differ in patient care practices. Further randomized controlled research is required to validate the results of this study.

## 5. Conclusions

In patients with moderate to severe ARDS who underwent prone positioning, post-prone positioning or change in MP, MP/PBW, and MP/compliance were significantly different between 28-day survivors and nonsurvivors, and post-prone positioning or change in MP/compliance was identified as an independent predictive factor for 28-day mortality. However, further randomized controlled research is required to elucidate the causality of decreased MP and improved clinical outcomes.

## Figures and Tables

**Table 1:** Demographic data of 28-day survivors and nonsurvivors.

Characteristics	Total Patients	Survival in 28 Days	Nonsurvival in 28 Days	*p* Value
	(*n* = 135)	(*n* = 91)	(*n* = 44)	
Age (years)	58.2 ± 13.0	57.2 ± 14.2	60.1 ± 9.9	0.166
Sex (male/female)	86/49	59/32	27/17	0.694
BMI (kg/m^2^)	25.6 ± 5.4	26.7 ± 5.8	23.4 ± 3.6	<0.001 *
Comorbidity				
Malignancy	21 (15.6%)	8 (8.8%)	13 (29.5%)	0.002 *
Chronic liver disease	12 (8.9%)	8 (8.8%)	4 (9.1%)	>0.999
Heart failure	3 (2.2%)	1 (1.1%)	2 (4.5%)	0.248
End-stage renal disease	10 (7.4%)	6 (6.6%)	4 (9.1%)	0.728
Diabetes Mellitus	48 (35.6%)	31 (34.1%)	17 (38.6%)	0.603
Chronic lung disease	4 (3.0%)	4 (4.4%)	0 (0.0%)	0.303
Chronic steroid usage	8 (5.9%)	4 (4.4%)	4 (9.1%)	0.437
Etiology of ARDS				0.012 *
Viral pneumonia	86 (63.7%)	63 (69.2%)	23 (52.3%)	
Bacterial pneumonia	43 (31.9%)	27 (29.7%)	16 (36.4%)	
Sepsis	6 (4.4%)	1 (1.1%)	5 (11.4%)	
Severity index				
APACHE II score	26.4 ± 7.2	24.9 ± 7.5	29.5 ± 5.5	<0.001 *
SOFA score	12.4 ± 3.1	11.9 ± 3.0	13.6 ± 2.8	0.003 *
Laboratory data				
White blood cell (1000/μL)	12.0 ± 16.0	10.4 ± 6.9	15.4 ± 26.2	0.264
C-reactive protein (mg/dL)	17.6 ± 10.6	15.4 ± 9.6	22.0 ± 11.3	0.001 *
Lactate (mg/dL)	24.2 ± 24.3	20.6 ± 18.7	31.0 ± 31.5	0.046 *
Albumin (mg/dL)	2.7 ± 0.6	2.8 ± 0.5	2.6 ± 0.7	0.216
Creatinine (mg/dL)	2.0 ± 2.5	1.6 ± 2.1	2.8 ± 3.1	0.004 *
Total bilirubin (mg/dL)	0.9 ± 1.2	0.9 ± 1.3	0.8 ± 0.8	0.303
Arterial blood gas				
pH	7.3 ± 0.1	7.3 ± 0.1	7.3 ± 0.1	0.701
PaO_2_/FiO_2_ ratio (mmHg)	87.5 ± 42.1	93.3 ± 46.4	75.4 ± 28.3	0.020 *
PaCO_2_ (mmHg)	51.5 ± 15.8	51.7 ± 16.7	51.1 ± 13.8	0.830
HCO_3_ (mmol/L)	25.5 ± 5.3	25.8 ± 5.5	25.1 ± 4.9	0.489
Interventions				
Period from ARDS onset to prone positioning (days)	1.7 ± 2.6	1.6 ± 2.8	2.1 ± 2.3	0.070
Duration of prone positioning (days)	3.5 ± 3.6	3.5 ± 3.8	3.5 ± 3.0	0.751
Fresh hemodialysis	37 (27.4%)	18 (19.8%)	19 (43.2%)	0.004 *
Muscle relaxant usage	134 (99.3%)	90 (98.9%)	44 (100.0%)	0.485
Vasopressor usage	91 (67.4%)	57 (62.6%)	34 (77.3%)	0.089
ECMO after prone positioning	18 (13.3%)	11 (12.1%)	7 (15.9%)	0.540

* *p* < 0.05. APACHE II: Acute Physiology and Chronic Health Evaluation II; ECMO: extracorporeal membrane oxygenation; FiO_2_: fraction of inspired oxygen; PaCO_2_: partial pressure of carbon dioxide in the arterial blood; PaO_2_: partial pressure of oxygen in the arterial blood; SOFA: sequential organ failure assessment.

**Table 2:** Ventilator parameters of 28-day survivors and nonsurvivors.

Characteristics	Survival	Nonsurvival	*p* Value
	(*n* = 91)	(*n* = 44)	
Before prone positioning			
PaO_2_/FiO_2_ ratio (mmHg)	93.3 ± 46.4	75.4 ± 28.3	0.020 *
pH	7.3 ± 0.1	7.3 ± 0.1	0.793
PaCO_2_ (mmHg)	51.7 ± 16.7	51.1 ± 13.8	0.830
Peak airway pressure (cmH_2_O)	31.9 ± 4.4	33.5 ± 5.4	0.730
Positive end expiratory pressure (cmH_2_O)	13.6 ± 3.2	13.3 ± 2.5	0.590
Tidal volume/predicted body weight (mL/Kg)	7.4 ± 1.7	7.4 ± 1.8	0.998
Respiratory rate (/min)	24.8 ± 4.5	24.5 ± 5.1	0.710
Dynamic driving pressure (cmH_2_O)	18.3 ± 3.9	20.2 ± 5.3	0.035 *
Dynamic compliance (mL/cmH_2_O)	24.0 ± 7.5	22.6 ± 9.6	0.251
Mechanical power (J/min)	23.3 ± 6.5	22.8 ± 5.7	0.688
Mechanical power/predicted body weight (10^−3^ J/min/kg)	407.0 ± 115.6	407.2 ± 121.7	0.992
Mechanical power/compliance (J/min/mL/cmH_2_O)	1.1 ± 0.4	1.2 ± 0.6	0.274
After prone positioning			
PaO_2_/FiO_2_ ratio (mmHg)	137.5 ± 64.0	108.9 ± 51.9	0.013 *
pH	7.4 ± 0.1	7.3 ± 0.1	<0.001 *
PaCO_2_ (mmHg)	47.2 ± 13.1	53.9 ± 15.7	0.011 *
Peak airway pressure (cmH_2_O)	31.0 ± 3.7	34.9 ± 5.1	<0.001 *
Positive end expiratory pressure (cmH_2_O)	14.2 ± 2.3	14.2 ± 2.4	0.950
Tidal volume/predicted body weight (mL/Kg)	7.2 ± 1.6	7.0 ± 1.4	0.402
Respiratory rate (/min)	24.9 ± 4.5	26.8 ± 4.3	0.017 *
Dynamic driving pressure (cmH_2_O)	16.8 ± 3.7	20.7 ± 5.3	<0.001 *
Dynamic compliance (mL/cmH_2_O)	25.7 ± 8.1	21.0 ± 8.5	0.002 *
Mechanical power (J/min)	22.6 ± 6.5	25.3 ± 6.2	0.024 *
Mechanical power/predicted body weight (10^−3^ J/min/kg)	396.9 ± 118.9	449.3 ± 118.8	0.018 *
Mechanical power/compliance (J/min/mL/cmH_2_O)	0.9 ± 0.3	1.4 ± 0.6	<0.001 *
Changes before and after prone positioning			
Change in mechanical power (J/min)	−0.6 ± 5.7	2.5 ± 7.4	0.007 *
Change in mechanical power/predicted body weight (10^−3^ J/min/kg)	−9.2 ± 97.5	42.1 ± 127.9	0.010 *
Change in mechanical power/compliance (J/min/mL/cmH_2_O)	−0.1 ± 0.3	0.2 ± 0.3	<0.001 *

* p < 0.05.

**Table 3:** Univariate and multivariate analysis results for 28-day mortality predictors.

	Univariate Analysis		Multivariate Analysis Model 1		Multivariate Analysis Model 2	
	Hazard Ratio (95% CI)	*p* Value	Hazard Ratio (95% CI)	*p* Value	Hazard Ratio (95% CI)	*p* Value
Sex						
Female	1 (reference)					
Male	0.875 (0.477–1.606)	0.667				
Age, per 1 year increment	1.013 (0.991–1.035)	0.263				
Body mass index, per 1 kg/m^2^ increament	0.894 (0.837–0.955)	0.001 *	0.926 (0.859–0.999)	0.046 *	0.928 (0.853–1.009)	0.081
Cormobidity: malignancy						
No	1 (reference)					
Yes	2.758 (1.440–5.281)	0.002 *	1.150 (0.472–2.803)	0.759	1.640 (0.786–3.422)	0.187
APACHE II score, per 1 increment	1.076 (1.030–1.123)	0.001 *	1.025 (0.964–1.089)	0.428	1.033 (0.971–1.099)	0.301
SOFA score, per 1 increment	1.171 (1.065–1.287)	0.001 *	1.086 (0.955–1.235)	0.206	1.172 (1.012–1.356)	0.034 *
Laboratory data						
C-reactive protein, per 1 mg/dL increment	1.047 (1.021–1.075)	<0.001 *	1.017 (0.986–1.048)	0.290	1.020 (0.989–1.052)	0.213
Lactate, per 1 mg/dL increment	1.010 (1.002–1.018)	0.020 *				
Creatinine, per 1 mg/dL increment	1.125 (1.034–1.225)	0.006 *	1.116 (0.983–1.267)	0.090	1.065 (0.939–1.209)	0.327
Blood gas analysis and respiratory mechanism before prone positioning						
PaO_2_/FiO_2_ ratio, per 1 mm Hg increment	0.987 (0.977–0.998)	0.017 *				
PaCO_2_, per 1 mm Hg increment	0.998 (0.980–1.017)	0.844				
Mechanical power, per 1 J/min increment	0.994 (0.948–1.042)	0.804				
Mechanical power/predicted body weight, per 1 10^−3^ J/min/kg increment	1.000 (0.998–1.003)	0.835				
Mechanical power/compliance, per 1 J/min/mL/cmH_2_O increment	1.645 (0.866–3.126)	0.129				
Blood gas analysis and respiratory mechanism after prone positioning for 1 day						
PaO_2_/FiO_2_ ratio, per 1 mm Hg increment	0.992 (0.985–0.998)	0.010 *	0.991 (0.985–0.998)	0.015 *	0.994 (0.987–1.001)	0.103
PaCO_2_, per 1 mm Hg increment	1.027 (1.008–1.047)	0.004 *	0.998 (0.972–1.025)	0.891	1.021 (0.998–1.043)	0.074
Mechanical power, per 1 J/min increment	1.049 (1.006–1.094)	0.027 *				
Mechanical power/predicted body weight, per 1 10^−3^ J/min/kg increment	1.003 (1.001–1.005)	0.017 *				
Mechanical power/compliance, per 1 J/min/mL/cmH_2_O increment	5.209 (2.972–9.129)	<0.001 *	3.486 (1.422–8.546)	0.006 *		
Change in mechanical power, per 1 J/min increment	1.050 (1.010–1.091)	0.013 *				
Change in mechanical power/predicted body weight, per 1 10^−3^ J/min/kg increment	1.003 (1.000–1.005)	0.017 *				
Change in mechanical power/compliance, per 1 J/min/mL/cmH_2_O increment	8.231 (3.895–17.395)	<0.001 *			7.972 (3.071–20.697)	<0.001 *

* *p* < 0.05. APACHE II: Acute Physiology and Chronic Health Evaluation II; CI: confidence interval; FiO_2_: fraction of inspired oxygen; PaCO_2_: partial pressure of carbon dioxide in the arterial blood; PaO_2_: partial pressure of oxygen in the arterial blood; SOFA: sequential organ failure assessment.

## Data Availability

The datasets used and/or analyzed during the current study are available from the corresponding author on reasonable request.
